# Decadal trends in Red Sea maximum surface temperature

**DOI:** 10.1038/s41598-017-08146-z

**Published:** 2017-08-15

**Authors:** V. Chaidez, D. Dreano, S. Agusti, C. M. Duarte, I. Hoteit

**Affiliations:** 1 King Abdullah University of Science and Technology (KAUST), Red Sea Research Center (RSRC), Thuwal, 23955-6900 Saudi Arabia; 2 King Abdullah University of Science and Technology (KAUST), Computer, Electrical and Mathematical Sciences and Engineering Division (CEMSE), Thuwal, 23955-6900 Saudi Arabia; 3 King Abdullah University of Science and Technology (KAUST), Physical Sciences and Engineering Division, Thuwal, 23955-6900 Saudi Arabia

## Abstract

Ocean warming is a major consequence of climate change, with the surface of the ocean having warmed by 0.11 °C decade^−1^ over the last 50 years and is estimated to continue to warm by an additional 0.6 – 2.0 °C before the end of the century^1^. However, there is considerable variability in the rates experienced by different ocean regions, so understanding regional trends is important to inform on possible stresses for marine organisms, particularly in warm seas where organisms may be already operating in the high end of their thermal tolerance. Although the Red Sea is one of the warmest ecosystems on earth, its historical warming trends and thermal evolution remain largely understudied. We characterized the Red Sea’s thermal regimes at the basin scale, with a focus on the spatial distribution and changes over time of sea surface temperature maxima, using remotely sensed sea surface temperature data from 1982 – 2015. The overall rate of warming for the Red Sea is 0.17 ± 0.07 °C decade^−1^, while the northern Red Sea is warming between 0.40 and 0.45 °C decade^−1^, all exceeding the global rate. Our findings show that the Red Sea is fast warming, which may in the future challenge its organisms and communities.

## Introduction

Ocean warming with climate change^1^ is creating challenges for organisms, which accommodate to warming by shifting their distribution poleward and advancing their phenology^2^. While parts of the ocean may be warming gradually, others may experience rapid fluctuations, tipping points, or extreme weather events, such as heat waves, likely inducing greater impacts on biodiversity^1, 3^, as exemplified by the impacts of heat waves on seagrass^4, 5^ and other organisms in the Mediterranean, a rapidly warming sea^6^. Extreme heat events such as ocean heat waves propagated by El Niño-Southern Oscillation are also major concerns for coral reefs as they may lead to bleaching^7–9^. The magnitude and duration of such events is important for organisms experiencing temperature anomalies outside their optimal thermal range and perhaps even above their thermal limits. High temperature anomalies of air and water are also linked to stratification of the water column, potentially diminishing oxygen levels and/or increasing microbial virulence, thus causing mass mortality of organisms and disrupting community structure^10–12^.

Impacts of warming are likely to be greatest in semi-enclosed seas, which tend to support warming rates faster than average^5, 13^ and where the capacity of organisms to adapt to warming by shifting their biogeographical range poleward is limited by the presence of continental masses^14^, rendering most semi-enclosed seas climatic sink areas for marine organisms^15^.

The Red Sea is a semi-enclosed, extremely warm sea basin, experiencing rapid warming^16–19^. Between 1982 and 2006, the average annual temperature of the Red Sea increased by 0.74 °C^17^, comparable to the global average of 0.85 °C^1^. An intense warming event occurred in 1994 leading to a 0.7 °C increase in mean annual SST (sea surface temperature)^18^. Modern average temperatures in the Red Sea already exceed those of other tropical regions^20, 21^. Although it is considered a fast warming, large marine ecosystem, its thermal regimes and evolution remain largely unresolved^17, 22^. Yet, the Red Sea hosts one of the largest reef systems in the world, where organisms may be already close to their thermal limits.

Whereas most analyses focus on mean seawater temperature, maximum temperature may be a more relevant property in relation to some specific questions. For instance, thermal collapse is determined by temperature exceeding the thermal capacity of organisms^23^, which is, therefore, dependent on the maximum, rather than the mean temperature the organisms experience. This may be particularly important in the Red Sea where maximum seawater temperatures are already extremely high. Yet, available analyses of thermal regimes in the Red Sea focus on annual mean values^18, 19, 24, 25^, rather than the dynamics of maximum temperature. Here we characterize the variability in temperature maxima across the Red Sea and over time (1982 to 2015), based on daily values, identifying rates of change in annual maximum sea surface temperature, hereafter T_max_, as well as the distribution of anomalies, relative to T_max_ over time.

## Results

### Warming rates and timing

**Figure 1:**
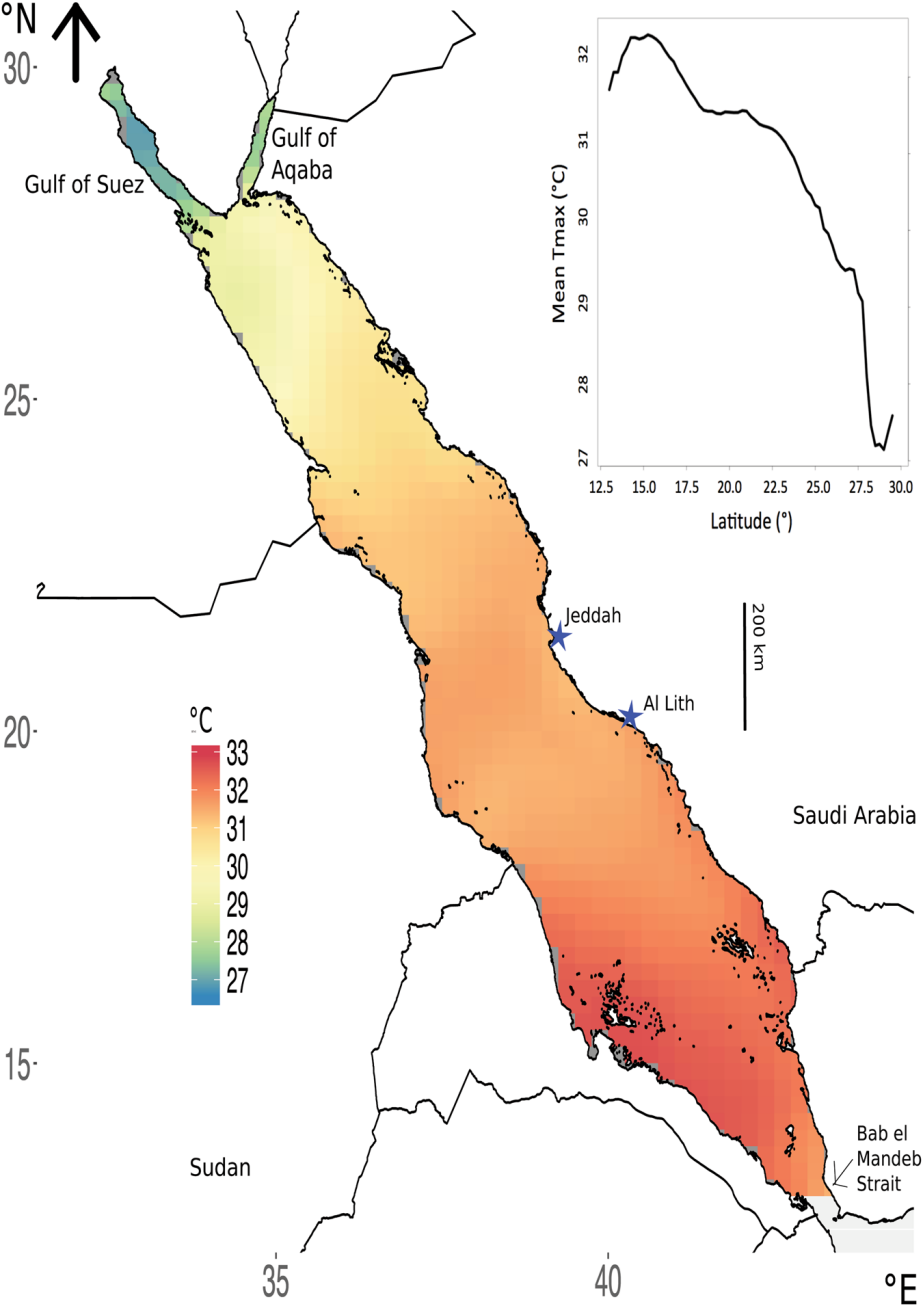
Distribution of mean (from 1982 to 2015) maximum annual temperature (T_max_) across the Red Sea. Insert shows the latitudinal changes in mean (from 1982 to 2015) T_max_. Values based on daily temperature data. Image created using R (v3.3.1, www.R-project.org)^45^ including packages: ggplot2^46^ and rasterVis^47^, RStudio (v1.0.143, www.rstudio.com), and InkScape (v0.91, www.inkscape.org).

The Red Sea displays a latitudinal gradient of increasing T_max_ from north to south with the southern Red Sea exhibiting the highest T_max_ (33 °C) until the southernmost Bab-el-Mandeb Strait (Fig. 1). The Gulf of Suez and the Gulf of Aqaba both exhibit lower temperatures than the open Red Sea (Fig. 1).

**Figure 2:**
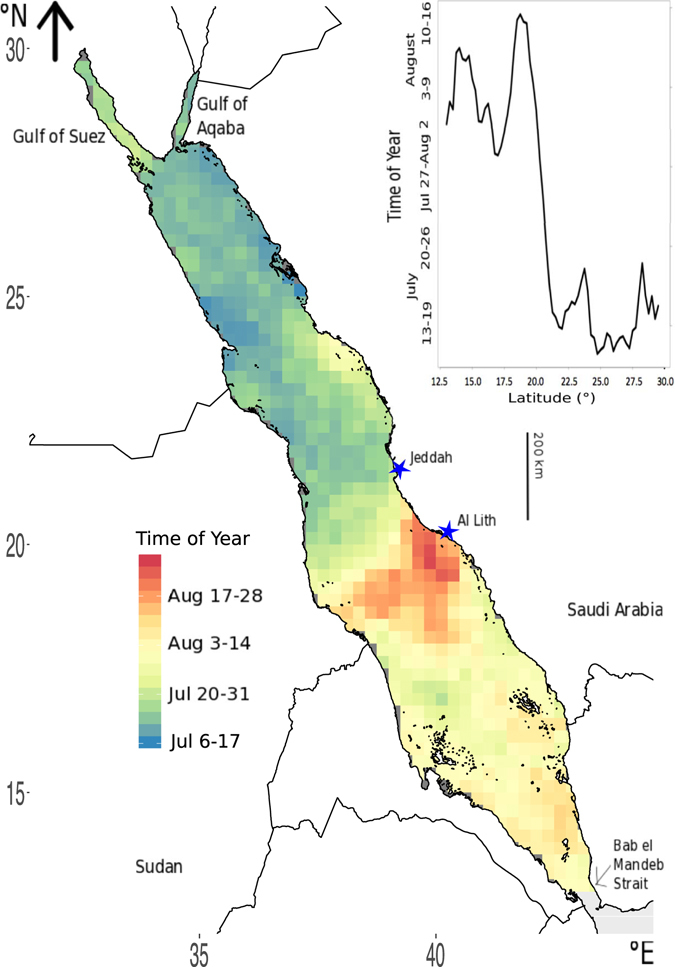
Average yearly timing of maximum annual temperature (T_max_) across the Red Sea. Insert shows the latitudinal trend in the average timing of T_max_. Image created using R (v3.3.1, www.R-project.org)^45^ including packages: ggplot2^46^ and rasterVis^47^, RStudio (v1.0.143, www.rstudio.com), and InkScape (v0.91, www.inkscape.org).

The northern Red Sea experiences T_max_ throughout July while T_max_ is reached between late July and mid–August in the southern Red Sea (Fig. 2). The area off of Al Lith, Saudi Arabia, prominently exhibits delayed T_max_ from approximately mid August to early September (red area in Fig. 2).

**Figure 3:**
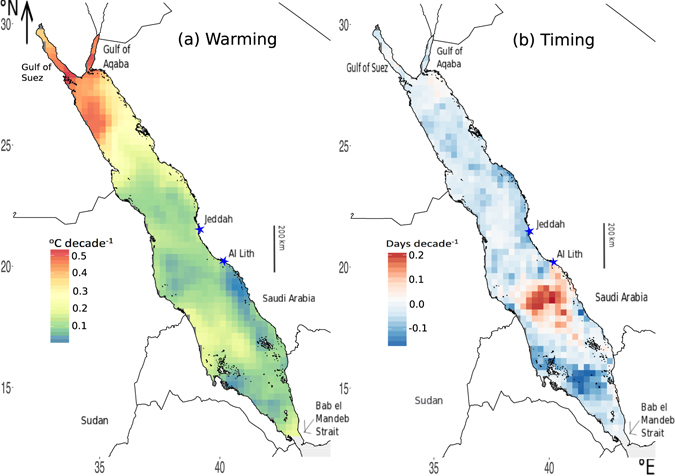
(**a**) Decadal rates of warming (°C decade^−1^) and (**b**) change in timing (days decade^−1^) of mean maximum annual temperature (T_max_) across the Red Sea. Image created using R (v3.3.1, www.R-project.org)^45^ including packages: ggplot2^46^ and rasterVis^47^, RStudio (v1.0.143, www.rstudio.com), and InkScape (v0.91, www.inkscape.org).

We assessed the rate of change in the magnitude and timing of T_max_ across the Red Sea. We observed a significant trend toward increased T_max_ across the Red Sea, at an average rate of 0.17 ± 0.07 °C decade^−1^ (p = 0.02, df = 32, t = 2.437). Rates of change in T_max_ varied across the Red Sea, with highest rates found in the colder areas of the Red Sea, including the northern Red Sea with rates for the Gulf of Suez and Gulf of Aqaba at 0.40 – 0.45 °C decade^−1^ (Fig. 3a). The region experiencing the lowest rate of warming is, again, that exhibiting a delayed T_max_ off the coast of Al Lith, Saudi Arabia (blue area in Fig. 3a).

In addition to a general pattern toward increasing T_max_, maximum temperatures in the Red Sea are also being reached earlier, with an average rate of change in the timing of T_max_ of 0.19 ± 0.30 days earlier decade^−1^ (Fig. 3b). Most of the Red Sea experienced progressively earlier T_max_ by 0.1 to 2 days earlier decade^−1^, but a region in the southern Red Sea showed a delay in T_max_ by 1 to 2 days decade^−1^. This is the same region that exhibits anomalous trends in the annual timing of T_max_ (Fig. 2).

### Heat anomalies

**Figure 4:**
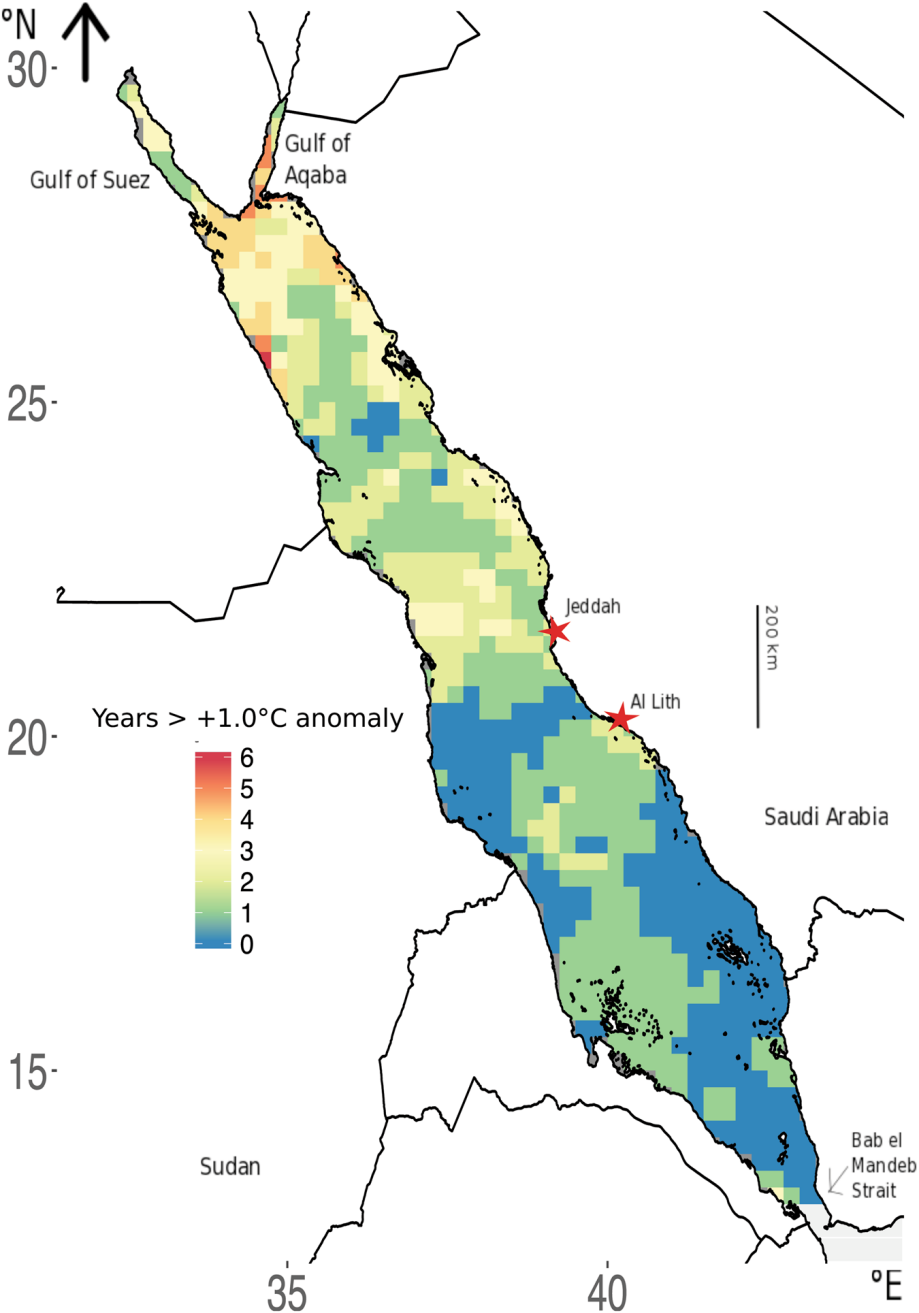
Distribution of the frequency, as number of years, across the Red Sea when maximum annual temperature (T_max_) reached 1.0 °C higher than the mean T_max_ for 1982–2015. Image created using R (v3.3.1, www.R-project.org)^45^ including packages: ggplot2^46^ and rasterVis^47^, RStudio (v1.0.143, www.rstudio.com), and InkScape (v0.91, www.inkscape.org).

Heat waves representing anomalies of 1.0 °C above the average T_max_ were observed more frequently in the northern half of the Red Sea over the last 34 years. The majority of the basin experienced such anomalies during at least one year and up to 6 years (which may or may not have been successive years). Some areas in the northern Red Sea, including the Gulf of Aqaba, experienced 1.0 °C magnitude heat waves as often as 5 or 6 years over the 34 year period examined here (Fig. 4).

**Figure 5:**
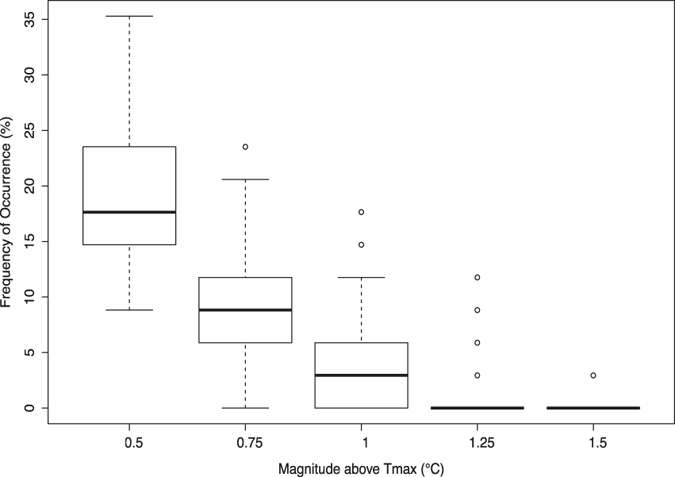
Probability, as the frequency of occurrence between 1982–2015, of maximum annual temperature (T_max_) anomalies of different magnitudes. A Kruskal-Wallis test and post-hoc Dunn’s tests found significantly different frequencies among and between all anomalies (Kruskal-Wallis, p < 2.2 e^−16^, chi-squared = 2674, df = 4; all Dunn’s tests, p < 0.05, Z range = [4:44]).

T_max_ values 0.5 °C above the mean (1982 – 2015) values occurred 15 to 24% of the years, whereas thermal anomalies involving T_max_ values 0.75 °C above the mean values occurred 6 to 12% of the years, and years with T_max_ values of 1.0 °C above the mean values occurred with a probability <6% (Fig. 5). The decline in the frequency of T_max_ anomalies with increasing magnitude of anomalies was significant (Kruskal-Wallis, p < 2.2 e^−16^, chi-squared = 2674, df = 4, Fig. 5) and significant differences were found among all groups (Dunn’s, p < 0.05, Z range = [4:44]).

## Discussion

The latitudinal gradient of increasing T_max_ from north to south in the Red Sea is largely a consequence of the variation in solar radiation associated with these latitudinal differences, and is consistent with previous studies reporting the same trend based on mean temperatures, with the warmest thermal regime in the southern region^19^. The Gulf of Suez and the Gulf of Aqaba have colder thermal regimes. Previous studies reported that, in the summer, the surface water entering the Gulf of Aqaba from the Red Sea is about 2 °C warmer than the water inside the Gulf^26^.

The Red Sea basin presents a discontinuity in terms of the timing of T_max_, associated with an abrupt transition between 20 and 22 °N. The timing of T_max_ occurs two months earlier south of this boundary compared to the timing north of this boundary. The distinct break between North and South (Fig. 2), may be evidence for the strong coupling of wind and sea surface temperatures over the basin as in other ocean systems^27–29^. During winter (October–April), the basin experiences opposing southward and northward winds, converging at about the same belt between 19 – 20 °N^19^ where the divide in timing of T_max_ is observed. From May to September, the major wind vector is from north to south^19^.

The warming rate of the Red Sea, 0.17 ± 0.07 °C decade^−1^, is higher than the global ocean rate of 0.11 °C decade^-1 1^. The northern Red Sea is warming faster with the Gulf of Suez and Gulf of Aqaba (0.40 – 0.45 °C decade^−1^) (Fig. 3a) warming four times faster than the mean global ocean warming rate. The semi-enclosed nature of the two gulfs as well as that of the Red Sea as a whole may account for the intense warming^17, 30, 31^, while the slower rate of increase in the southern Red Sea may be buffered by its closer connection to the Indian Ocean. Although the northern Red Sea is warming faster, it remains the coolest region in the basin throughout the year.

**Figure 6:**
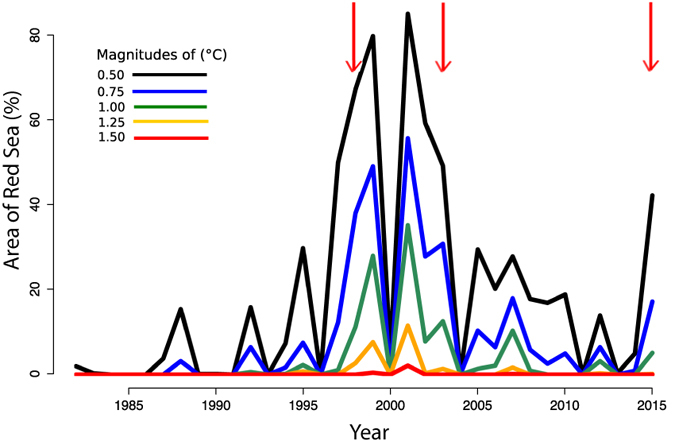
Percent of Red Sea area exhibiting maximum annual temperature (T_max_) anomalies of different magnitudes between 1982 and 2015. Red indicators signal the occurrence of El Niño events.

Increased T_max_ will have effects on marine biota, which are particularly vulnerable to heat waves, when their thermal limits may be approached or exceeded^23, 32^. The occurrence of heat anomalies, which are also likely to increase in the future^1^, are greatly relevant to the physiology of organisms, particularly for those inhabiting already warm environments, like the Red Sea, where temperature anomalies may lead to thermal collapse^24, 32–34^. The years 1999 and 2001 experienced the largest anomalies across the basin (Fig. 6). During the years 1997 – 1998, one of the strongest El Niño events occurred, while 2000 – 2001 was considered a weak La Niña event^35^. The years 2003 and 2015, also El Niño years, showed the second greatest percentage of area covered by T_max_ anomalies, although of a relatively small, 0.5 °C, magnitude (Fig. 6).

Systematic monitoring efforts are required to detect the effect of heat anomalies on marine organisms, such as bleaching and mass mortality events^36^. Unfortunately, there is no systematic monitoring of biological events in the Red Sea, such as bleaching events, which may be affected by thermal anomalies such as those reported here. Extensive bleaching was reported in the southern half of the Red Sea in 2015, one of the years with extensive, but relatively moderate, thermal anomalies in our analysis (Fig. 6). Whether bleaching events also occurred in other years with extensive T_max_ anomalies is unknown due to lack of long-term monitoring.

The distribution of T_max_ in the Red Sea conforms to the four provinces, described by Raitsos *et al*.^19^ based on phytoplankton biomass. The warmer T_max_ regime in the South is associated with higher phytoplankton biomass, while the lowest T_max_ in the northern Red Sea is associated with the lowest phytoplankton biomass. However, this pattern may be a result of the decrease in nutrient concentrations from south to north along the Red Sea^37^, rather than its thermal regime. A region in the central Red Sea emerges as deviating from the general pattern with a slower rate of warming and T_max_ reached later in the year over time.

That T_max_ is rapidly increasing in the Red Sea, which is already one of the warmest seas, anticipates challenges to biota. Whereas T_max_ is increasing more rapidly in the North than in the South, the warmer thermal regime in the South may already be near the thermal limits of organisms and, therefore, even a modest increase in T_max_ may suffice to exceed their thermal tolerance, although experimental work is necessary to test this suggestion. Unfortunately, although the Red Sea ranks as the warmest sea on the planet, aside from one study examining the effect of temperature on grazing rates of Red Sea parrotfish^38^, there is, at present, no quantitative information on the thermal limits of Red Sea biota. However, reports of a decline in coral growth and calcification across the thermal range of Red Sea corals^39^, together with widespread bleaching in the southern half of the Red Sea during 2015, as well as lower growth rates reported for brown macroalgae^40^, suggests that warm Red Sea temperatures already challenge the capacities of organisms. In addition to increasing T_max_, the general tendency towards an earlier occurrence indicates that phenology patterns of organisms might need to adjust to this shift. Marine organisms generally cope with warming by shifting their biogeographical range poleward tracking the migration of isotherms^2, 14^. However, this strategy is not possible in semi-enclosed seas, such as the Red Sea^14, 15^, rendering its large pool of endemic species at risk of extinction unless they become Lessepsian migrants and colonize the Mediterranean Sea as a hundred Red Sea species have done^41^. Altogether, higher and earlier T_max_ may challenge the capacities of Red Sea biota to cope.

Results presented here provide a context for experimental analyses examining thermal limits, by defining the regimes and trends in T_max_ across the Red Sea, as well as the likelihood of observing anomalies of different magnitudes. In addition, these results may help understand biodiversity patterns and losses across natural gradients in the Red Sea by matching the distribution of communities and habitats with the distribution of T_max_. This will provide an underpinning to the assessment thermal maxima play in explaining patterns of biodiversity across the Red Sea.

In conclusion, Red Sea biota are exposed to increased ocean warming, particularly in the northern Red Sea, which may affect their future persistence, especially if unable to migrate into the Mediterranean. The results on Red Sea warming presented here, coupled with experimental evidence on the thermal limits of Red Sea organisms, yet to be resolved, would provide a powerful tool to predict the future of marine biodiversity in this biodiversity hotspot containing a high degree of endemism.

## Methods

### The dataset

We used remotely sensed sea surface temperature (SST, °C) data to examine maximum temperatures on a basin-wide scale across the Red Sea. The AVHRR–OI (Advanced Very High Resolution Radiometer–Optimum Interpolation) Pathfinder sensor currently provides the longest continuous daily dataset of infrared SST from 1981 to present^42^, allowing the assessment of decadal trends of temperatures. Whereas other sensors provide higher resolution, in terms of pixel size, they encompass a period too short to be climatically-relevant as yet (ERS-1/ATSR-1 and Acqua/AMSR-E)^43^ and do not allow us to identify, with confidence, the maximum temperature achieved over time. A daily Level-4, gap-free dataset merging day and night analysis AVHRR SST was obtained from NASA’s (National Aeronautics and Space Administration) National Climatic Data Center^44^ at podaac.jpl.nasa.gov accessed on January 5, 2016 encompassing 34 years over the period 1982 to 2015. This dataset has been optimally interpolated and mapped on a 0.25° × 0.25° grid. The values in the dataset were corrected with *in situ* measurements from buoys and ships^42^. Daily fluctuations in daily SST time series may significantly affect the measurement of maximum SST phenology and magnitude, because the recurrence of the passage of AVHRR Pathfinder is 2 to 3 days and, the time of passage may not match the time of T_max_, typically found in the late afternoon with a daily range in T_max_, derived from moorings in the central Red Sea, of up to 3 °C. Moreover, the individual estimates may be affected by dust, which is prevalent in the region at the time of T_max_, and cloud cover. Whereas the data we used is interpolated, the individual daily values may be affected by the sources of error above, leading to underestimates of the actual T_max_. To attenuate this source of error, we extracted the maximum daily T value within sets of interpolated daily values over 8-day periods, and then selected, for each of the 669 pixels, the highest T observed in any one year as that providing the best estimate of T_max_ for that pixel and year. The dataset can be downloaded from the Pangea open-access data repository (Chaidez et al. 2017)^48^.

### Calculating decadal trends

The decadal trends of maximum temperatures and time of occurrence were estimated by fitting a linear regression relating T_max_ to year for each of the pixel’s yearly time series. The slopes of the fitted linear regressions provide an estimate of the rates of change for each pixel in the Red Sea (units: °C decade^−1^, and days decade^−1^, respectively). We tested the possible occurrence of autocorrelation in T_max_ among years, and found, for a sample of pixels, no evidence of autocorrelation, i.e. the T_max_ in any one year is independent of T_max_ in preceding year(s).

### Calculating heat anomalies

For each pixel, a reference maximum temperature was computed by taking the mean of the highest temperatures per year over the study period. A heat wave event was defined as a yearly maximum temperature above the reference maximum temperature by a given threshold chosen at 0.5 °C intervals between 0.5 and 1.5 °C. The number of heat wave events over the 34 years were counted for each pixel, as well as the area of the Red Sea experiencing heat waves of various magnitudes in a given year. A Kruskal-Wallis test followed by Dunn’s test for multiple comparisons, was used to compare the frequencies of occurrence for all magnitudes of heat anomalies in Fig. 5. The percentage of area in Fig. 6 was calculated as the percentage of pixels. We are aware that the area of each pixel depends on latitude, as the length of a degree longitude varies with latitude. However, for the narrow range of latitude covered by the Red Sea, the difference is minimal, so percent of pixels and area are essentially equivalent.

All data manipulation and analyses were conducted using R (v3.3.1, www.R-project.org)^45^.

### Data Availability

The data set supporting the analysis presented here can be found in the Pangaea open data repository: (Chaidez *et al.* 2017, http://www.pangaea.de)^48^.
