# Author Correction: Decadal trends in Red Sea maximum surface temperature

**DOI:** 10.1038/s41598-018-25731-y

**Published:** 2018-05-09

**Authors:** V. Chaidez, D. Dreano, S. Agusti, C. M. Duarte, I. Hoteit

**Affiliations:** 1 King Abdullah University of Science and Technology (KAUST), Red Sea Research Center (RSRC), Thuwal, 23955-6900 Saudi Arabia; 2 King Abdullah University of Science and Technology (KAUST), Computer, Electrical and Mathematical Sciences and Engineering Division (CEMSE), Thuwal, 23955-6900 Saudi Arabia; 3 King Abdullah University of Science and Technology (KAUST), Physical Sciences and Engineering Division, Thuwal, 23955-6900 Saudi Arabia

Correction to: *Scientific Reports* 10.1038/s41598-017-08146-z, published online 15 August 2017

In Figure 3b, the ‘Days decade^−1^’ scale from −1.0 to 2.0 is incorrectly given as from −0.1 to 0.2. The correct Figure 3 appears below as Figure 1.

**Figure 1:**
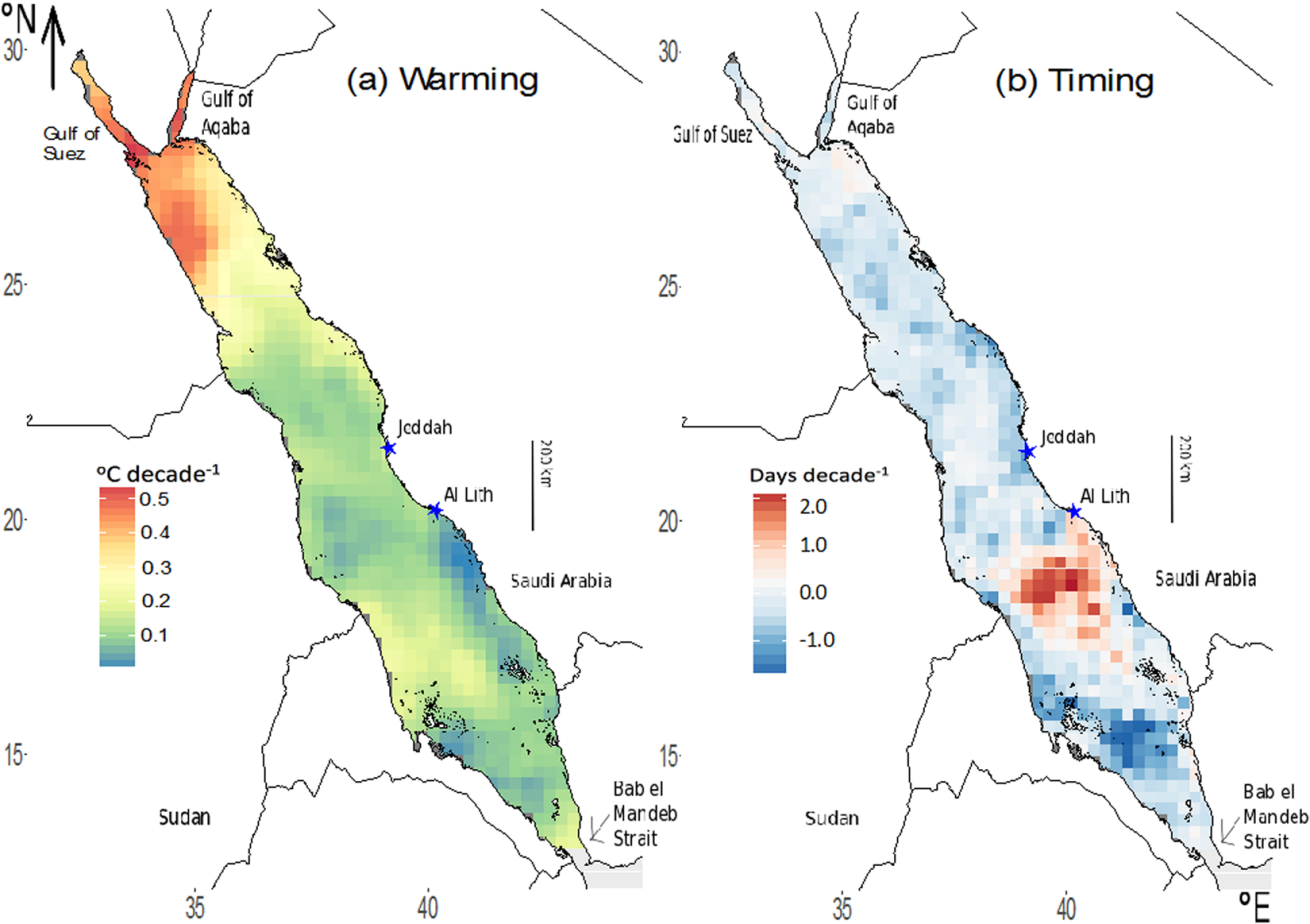
(**a**) Decadal rates of warming (°C decade_−1_) and (**b**) change in timing (days decade_−1_) of mean maximum annual temperature (T_max_) across the Red Sea. Image created using R (v3.3.1, www.R-project.org)^45^ including packages: ggplot2^46^ and rasterVis^47^, RStudio (v1.0.143, www.rstudio.com), and InkScape (v0.91, www.inkscape.org).

